# Learning curve for robotic thyroidectomy using BABA: CUSUM analysis of a single surgeon’s experience

**DOI:** 10.3389/fendo.2022.942973

**Published:** 2022-08-31

**Authors:** Hui Ouyang, Wenbo Xue, Zeyu Zhang, Rong Cong, Botao Sun, Fada Xia, Xinying Li

**Affiliations:** ^1^ Department of General Surgery, Xiangya Hospital, Central South University, Changsha, China; ^2^ Department of Cardiothoracic Surgery, The Third Xiangya Hospital of Central South University, Changsha, China

**Keywords:** robotic thyroidectomy, learning curve, neck dissection, papillary thyroid carcinoma, cumulative summation analysis

## Abstract

**Background:**

This study assessed the safety and oncologic outcomes of robotic thyroidectomy *via* the bilateral axillary breast approach (BABA RT) for conventional open procedures. The learning curves of BABA RT were further evaluated.

**Methods:**

An exact 1:1 matching analysis was performed to compare the technical safety and oncologic outcomes between robotic thyroidectomy and conventional open surgery. Learning curves were assessed using cumulative summation analysis.

**Results:**

There was no significant difference in general characteristics, short time outcomes (including transient hypoparathyroidism, transient postoperative hoarseness, hematoma/seroma, mean postoperative hospital stay, and other complications), the number of retrieved central lymph nodes, and recurrence rates between robotic BABA and conventional groups. The mean number of retrieved lateral LNs in the robotic group was significantly less than those in the conventional group. The learning curve for working space making, robotic lobectomy, and total thyroidectomy are approximately 15, 30, and 20 cases, respectively. No differences except for operation time were found between the learning group and the proficient group.

**Conclusions:**

Robotic thyroidectomy and neck dissection *via* BABA are feasible in terms of surgical completeness, surgical safety, and oncological safety. Our results provide a criterion for judging whether the surgeon has entered the stable stage of robotic thyroidectomy *via* BABA in terms of the operative time.

## Introduction

Conventional open thyroidectomy has traditionally been accepted as the standard surgery for thyroid diseases. It has been demonstrated that the noticeable scar on the anterior neck caused by open surgery can negatively affect patients’ quality of life, regardless of the perceived severity of the scar (1, 2). Due to the increasingly cosmetic requirements, especially in women, a variety of endoscopic extracervical approaches have been developed (3). Extracervical techniques have shown considerable evolution with approaches that have included transaxillary, breast, postauricular, and transoral routes (4). More and more studies provided strong evidence that endoscopic approaches are equally safe and effective treatments with additionally better cosmetic satisfaction for thyroid cancer as open procedures (5). However, there has also been a varied evidence base for each of these approaches with regard to technical feasibility, safety, patient satisfaction, and cost-effectiveness (4). Several technical difficulties and limitations in the application of endoscopic thyroidectomy have also been reported (6–8). For instance, in breast approaches, central lymph dissection (CLD) is restricted by claviculate. It will be difficult in superior thyroid artery dissection and left recurrent laryngeal nerve identification in oral approaches.

Robotic-assisted thyroid surgery has gained increased popularity worldwide with the introduction of the da Vinci Robot. Robotic approaches from remote sites include trans-axillary (9), bilateral axillary breast approach (BABA) (10), face-lift (11), transoral (12) and hybrid approaches. Robotic instruments have shown additional superiorities, which include tremor-free, stabilized and 7°freedom movement of instrument, three-dimensional endoscopic view, self-controlled traction, and optimized ergonomics, over other endoscopic instruments. Delicate anatomy operation with versatile instruments could assist in dealing with complex intraoperative situations, as well as lead to an easier preservation of the parathyroid gland and identification of nerves, vessels, and the branch of the thoracic duct. However, reports of improved postoperative outcomes and patient satisfaction have been in contrast to the financial burden, longer operative time, and increased training required, which, to date, have limited widespread application (4). After being proficient in endoscopic thyroidectomy and neck dissection with more than 10 years’ experience, Professor Li started to perform robotic thyroidectomy *via* the BABA (BABA RT). We aimed to compare the safety and oncologic outcomes of BABA RT to those for conventional open procedures in this study. The learning curves of BABA RT performed by a single experienced surgeon in our department were further evaluated to investigate how many cases are needed before a surgeon becomes proficient in performing this procedure. We present the following article in accordance with the Transparent Reporting of Evaluationswith Nonrandomized Designs (TREND) reporting checklist.^1^

## Methods

### Patients’ cohort

A total of 134 consecutive patients were enrolled with thyroid benign and malignant nodules underwent BABA RT by a single surgeon at the Department of General Surgery, Xiangya Hospital, Central South University, between March 2020 and June 2021. Professor Xinying Li is proficient in conventional open thyroid surgery, as well as in endoscopic thyroid surgeries *via* total mammary areolas and transaxillary and transoral approaches but had no prior experience with robotic surgery. The operative indications included: malignant tumor size <2 cm (without gross extrathyroidal extension); metastatic lymph nodes in central or lateral compartment were not fused with each other or fixed in the neck and benign nodules under 6 cm. An exact 1:1 matching analysis was performed to compare the technical safety and oncologic outcomes between robotic thyroidectomy and conventional open surgery. Patients were matched using the following criteria: age ( ± 3 years), tumor stage (exact match), tumor size ( ± 0.3 cm for malignant nodules and ±1 cm for benign nodules), and Hashimoto’s thyroiditis (exact match) (13). Medical records were reviewed retrospectively. The follow up times were 3–15 months. This study was reviewed and approved by the Ethics Committee of Xiangya Hospital, Central South University (No. 202108136). Informed written consent was obtained from the enrolled patients.

### Robotic operative procedures

**Figure 1:**
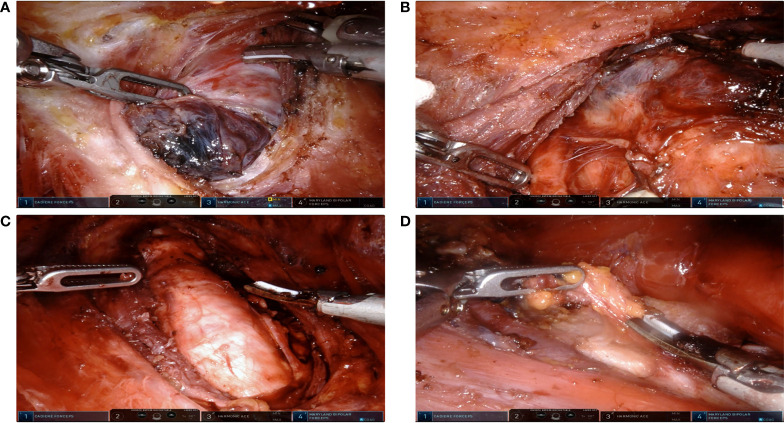
Extent of robotic surgeries. **(A)** Lobectomy due to benign thyroid nodules. **(B)** Lobectomy and ipsilateral Central Neck Dissection (CND) due to Papillary Thyroid Carcinoma (PTC). **(C)** Total thyroidectomy and ipsilateral CND due to PTC. **(D)** Total thyroidectomy with ipsilateral CND and lateral neck dissection. SCM were split longitudinally between its sternal head and clavicular head.

In brief, the patient was placed in a supine position with slight neck extension under general anesthesia. An outline of the flap area was drawn for the flap dissection. Skin flaps were raised by the injection of diluted epinephrine (1:200,000, 50 ml) fixed with ropivacaine (10 ml) in the subplatysmal space of the flap area, followed by blunt dissection. Two 8-mm axillary skin incisions and two 8-mm superomedial circumareolar incisions were then made. Four 8-mm Trocars were inserted after the flaps were raised, then connected with four robotic arms to finish robotic system docking. The instruments used were as follows: right circumareolar port for camera, left circumareolar port for Harmoic ACE scalpel, and bilateral axillary ports for Maryland and forceps. CO_2_ insufflation at 6–9 mm Hg was used to maintain the working space. The skin flap is extended at the neck superiorly to the thyroid cartilage (to the mandible for lateral neck dissection), posteriorly to the posterior border of SCM muscle. After the division of the midline, the thyroid isthmus was divided. The middle thyroid vein, superior thyroid artery, and inferior thyroid artery was then divided and ligated in order. The superior parathyroid glands and recurrent laryngeal nerve were preserved. The inferior parathyroid glands were preserved *in situ* if possible; otherwise, they were planted in SCM. Unilateral central compartment lymph node clearance was conducted including the prelaryngeal, pretracheal, and ipsilateral paratracheal areas. The lateral cervical compartment was exposed by splitting the SCM longitudinally and separation between the strap muscles. Dissected lateral neck compartment includes level III and IV lymph nodes (**Figure 1**). The techniques were the same as that previously described by other studies in detail (10, 14–16).

### Statistical analysis

Cumulative summation (CUSUM) analysis was designed for the quantitative estimation of the learning curve (plotting the operation time and determination of the case number to achieve mastery) as described (17). The 134 cases were ordered chronologically; the difference between the operative time of the *n*th case and the mean operative time (including docking time, lobectomy, total thyroidectomy, and neck dissection time) was defined as S_n_. S_n_ values were summed and plotted using the equation CUSUM= ∑S_n_. The slope of the CUSUM curve represents the trend of learning outcomes, and the point at which the slope changes from positive to negative is regarded as the point of overcoming the learning curve (17, 18). Continuous variables were presented as mean ± SD and mean (range). Continuous data were compared using Students’ t-test and one-way ANOVA, and dichotomous data were compared using chi-squared tests and Fisher’s exact tests through the SPSS 26.0 software. A *P*-value < 0.05 was considered statistically significant.

## Results

An exact 1:1 matching yielded 134 patients in the conventional open group as well as that in the robotic BABA group. The clinicopathological characteristics of the two groups are listed in **Table 1**. The robotic cohort consisted of 116 female and 18 male patients, and 36.4 (range 20–64). Eight patients underwent lobectomy due to benign thyroid nodules. Seventy-three patients underwent lobectomy and ipsilateral CND; 30 patients underwent total thyroidectomy and ipsilateral CND (20 patients for bilateral CND) due to papillary thyroid cancer. Additional lateral neck dissection (selective levels) was conducted in 11 patients (**Table 1** and **Figure 1**). No case was converted to open surgery. The two groups were similar in terms of age, gender ratio, tumor size, and Hashimoto’s thyroiditis ratio. There was no significant difference in transient hypoparathyroidism, transient postoperative hoarseness, hematoma/seroma, mean postoperative hospital stay, and other complications between two groups. Paresthesia of the chest wall in the robotic group was observed in 40 (29.8%) cases, which were significantly decreased in the third-month (10, 7.5%) visit in all the patients. However, the mean operating time was longer in the robotic group than in the conventional open group in all the surgical scopes. No significant difference was observed in the mean total number of retrieved central LNs (including the ipsilateral central compartment and bilateral central compartment). The mean number of retrieved lateral LNs in the robotic group was significantly less than those in the conventional group. This may be due to the fact that the extension of dissected levels in the robotic group only included selected level III and IV. During the follow-up period, no recurrence was observed in either group. Mean Tg levels (in patients with total thyroidectomy) in 1-month and 3-month follow-up points were not significantly different between the two groups.

**Table 1:** Demographic data and surgical outcomes between robotic group (bilateral axillary breast approach) and conventional open group.

Variables	BABA group (n = 134)		Open group (n = 134)		*p*- value*
Age, mean ± SD	36.40 ± 9.11		36.45 ± 8.61		0.962
Gender (male/female)	18/116		18/116		1.000
Hashimoto’s thyroiditis	18/116		18/116		1.000
Tumor size (mm)	10.31 ± 7.20		10.30 ± 6.48		0.996
Benign/Malignant	8/126		8/126		1.000
Extent of surgery					0.947
LT + ipsilateral CND	73		73		
TT + ipsilateral CND	30		27		
TT + bilateral CND	20		23		
TT + CND + LND	11		11		
Operation time (min)					
Docking	27.13 ± 9.82		NA		NA
LT + CND	81.75 ± 22.60		56.93 ± 10.83		<0.001
TT + CND**	116.67 ± 22.56		91.90 ± 40.14		<0.001
Transient hoarseness	5		4		0.735
Permanent hoarseness	0		0		NA
Transient hypoparathyroidism	20		31		0.093
Permanent hypoparathyroidism	1		2		0.562
Hematoma/seroma	1		0		NA
Paresthesia of chest wall
1 month after surgery	40		0		NA
3 months after surgery	10		0		NA
Retrieved lymph					
Ipsilateral central compartment	5.47 ± 4.20		5.23 ± 3.42		0.694
Bilateral central compartment	7.80 ± 4.85		8.25 ± 4.50		0.684
Lateral compartment	9.27 ± 6.93		24 ± 11.76		0.002
Postoperative hospital stays	2.49 ± 0.74		2.16 ± 0.39		0.328
Recurrence	0		0		NA
Tg levels (TT)					
1 month after surgery	1.02 ± 4.24		0.79 ± 2.13		0.735
3 months after surgery	0.41 ± 1.18		0.40 ± 0.95		0.978

TT, total thyroidectomy; LT, lobectomy; CND, central neck dissection; LND, lateral neck dissection; PGs, parathyroid glands; NA, not available. *Patients with censored dates were excluded. **Time involved in LND was not included.

Prophylactic or therapeutic unilateral (ipsilateral) CND was performed in all the cases. If necessary, bilateral CND and lateral neck dissection were performed. Lobectomy time was defined as the time from making a skin incision to lobectomy with ipsilateral CND. For cases with total thyroidectomy, the time of lobectomy can also be calculated separately. Therefore, all 134 cases have been enrolled for a lobectomy time study. The time of total thyroidectomy involved making a skin incision to total thyroidectomy with ipsilateral or bilateral CND (61 cases were enrolled). The docking time was also studied to understand the assistant’s (Hui Ouyang) learning curve.

**Figure 2:**
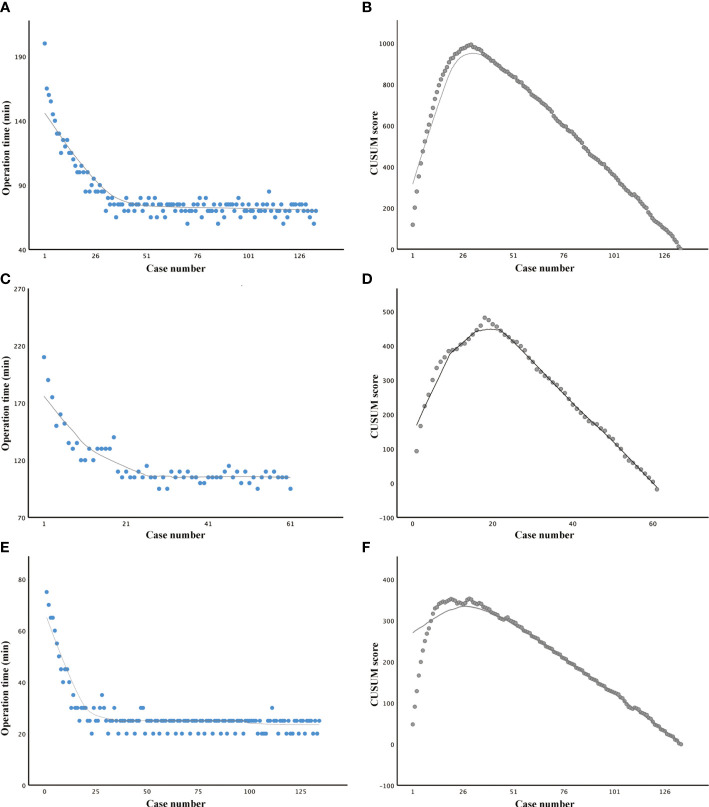
Learning curve studies of robotic thyroid surgery *via* the bilateral axillary breast approach. **(A, B)** Operation time plotted in chronological order and cumulative summation (CUSUM) test in patients who underwent lobectomy. **(C, D)** Operation time plotted in chronological order and CUSUM test in patients who underwent total thyroidectomy. **(E, F)** Operation time plotted in chronological order and CUSUM test in work spacing making.

The total operative time for lobectomy, on average, was 81.75 min. The slope of the learning curve shows that there was a peak at the 30th case, indicating that the learning curve for robotic lobectomy *via* BABA was 30 cases (**Figures 2A, B**). The total operative time for total thyroidectomy, on average, was 116.67 min. The slope of the learning curve shows that there was a peak at the 20th case, indicating that the learning curve for robotic total thyroidectomy *via* BABA was 20 cases (**Figures 2C, D**). The learning curve of robotic total thyroidectomy was much steeper than that of lobectomy because there was already a cumulative experience of over 20 cases of lobectomies before the completion of total thyroidectomy. The curve (with patients who underwent total thyroidectomy) was divided into two groups: the learning group (cases 1–20) and proficient group (cases 21–61). The demographic and surgical outcomes have been compared between these two groups. The operation time in the proficient group was significantly shorter than that in the learning group (105.24 VS 140.1 min). Other demographic, clinicopathological characteristics and complication rates showed no differences between the two groups (**Table 2**). Additionally, the docking time, on average, was 27.13 min. The slope of the learning curve shows that there was a peak at the 20th case, indicating that the learning curve for the assistant to dock the robotic system was 20 cases (**Figures 2E, F**).

**Table 2:** Demographic data and surgical outcomes between learning group and the proficient group under total thyroidectomy.

Variables	Learning group (n = 20)	Proficient group (n = 41)	*p*-value*
Age, mean ± SD	37.00 ± 9.96	36.1 ± 9.32	0.730
Gender (male/female)	3/20	5/41	0.761
Hashimoto’s thyroiditis	4/20	6/41	0.595
Tumor size (mm)	10.37 ± 5.21	11.88 ± 6.33	0.360
Extent of surgery			NA
TT+ ipsilateral CND	9	21	
TT+ bilateral CND	8	12	
TT+ CND + LND	3	8	
Operation time (mins)	140.10 ± 26.40	105.24 ± 4.87	< 0.001
Transient hoarseness	1	2	1.000
Permanent hoarseness	0	0	NA
Transient hypoparathyroidism	8	12	0.402
Permanent hypoparathyroidism	1	0	NA
Hematoma/seroma	0	0	NA
Paresthesia of chest wall			
1 month after surgery	6	12	0.953
3 months after surgery	2	3	1.000
Retrieved central compartment lymph	7.70 ± 6.95	7.61 ± 6.13	0.959
Postoperative hospital stays	2.25 ± 0.44	2.41 ± 0.63	0.300
Recurrence	0	0	NA
Tg levels (TT)			
1 month after surgery	0.65 ± 1.52	0.52 ± 1.64	0.770
3 months after surgery	0.43 ± 1.16	0.32 ± 1.04	0.707

TT, total thyroidectomy; CND, central neck dissection; NA, not available. *patients with censored dates were excluded.

## Discussion

The incidence of thyroid cancer has increased worldwide during the last decade, becoming the most common endocrine malignancy and accounting for 3.8% of new cancer diagnosis. Surgical resection, namely, conventional thyroidectomy, remains at the frontline of therapy, as surgical outcomes are undoubtedly successful (19). The conventional open approach leaves a neck scar that could be worrying mainly for young women. The recent progress in surgical technology, as well as patient cosmetic requests, has led to the development of alternative access to the thyroid lodge (20). Many remote-access approaches to the thyroid have been described to circumvent anterior neck scarring, including the transaxillary, BABA, robotic facelift, and transoral endoscopic vestibular approaches (21). BABA RT is one of the most popular remote-access approaches for thyroid surgery (22). Several studies have revealed that BABA robotic completion thyroidectomy could be performed safely without completion-related complication (22, 23). The experiences of utilizing BABA RT in lateral neck dissection have been shared in a few studies (10). In our study, no case under BABA RT was converted to open surgery. The complication rates and retrieved central and lateral neck lymph nodes were the same between robotic and open groups. No recurrence was observed in either group. These results revealed that BABA RT is feasible in terms of surgical completeness, surgical safety, and oncological safety. However, like other robotic approaches, its disadvantage is also obvious, adding extra cost.

The learning curve seems to be a more scientific estimation system for the perioperative period, which should include the appropriateness of the chosen surgical indications, rationality of the procedural steps, quality of manipulation, recovery of the patient, and postoperative quality of life (24). The learning curve for transaxillary robotic thyroidectomy is generally considered to be less steep; the reason for this may be that transaxillary access requires a profound understanding of the neck anatomy from a lateral to medial aspect (25). The transoral robotic approach has been reported to be as safe as the transoral endoscopic approach; however, no study regarding its learning curve has been reported (26). Kim reported that the learning curve for robotic thyroidectomy with CND using BABA was 40 cases for beginner surgeons (27). In another study about BABA RT, CUSUM analysis revealed that it took 50 cases for the surgeon to significantly improve the operation time (17). In our experience, we observed a shortening of the operative time after the first 30 cases. The surgeons involved in these studies regarding the BABA RT learning curve had none or little experience with endoscopic surgery, while the numbers of cases needed to achieve the learning curve are comparable with that in this study. This may be due to the fact that a previous experience with non-robotic endoscopic thyroidectomy or minimally invasive video-assisted thyroidectomy does seem to accelerate the learning curve of robotic thyroidectomy (25). Our results provide a criterion for judging whether the surgeon has entered the stable stage of BABA RT in terms of the operative time.

The learning curve for a particular procedure can be defined as the number of cases required to stabilize and minimize the operation time and complications (18). In a learning curve study, Wang et al. revealed that an operation time lasting more than 150 min and operation before proficiency were the risk factors of recurrent laryngeal nerve injury (28). In Liang’s study, a total of 90 consecutive patients who underwent BABA RT were enrolled. Based on the CUSUM results, they divided the patients into two phases: phase 1 (1–30 cases) and phase 2 (31–90 cases). The operative time, drainage amount, and blood loss decreased significantly in the phase 2 compared with phase 1. These results indicated that aside from the operative time, other complication factors may also serve as a surrogate for the learning curve evaluation (29). Kim et al. have studied the cumulative sums of transient hypoparathyroidism incidence in patients who underwent BABA RT. The incidence of hypoparathyroidism was 52.0% for the first 50 cases, which decreased to 46.0% for the 51st to the 100th case, and to 40.3% for the 101st to the 172nd case. CUSUM analysis indicated that the surgeon was proficient at the 75th case (17). In our study, the incidence of complications was low, no difference was found between the learning group (cases 1–20) and the proficient group (cases 21–61). This result shows that the reduction in complications does not need to reach the learning curve, which we speculate is related to Dr. Li’s experience in open and various kinds of endoscopic approaches before performing robotic surgeries.

## Conclusions

Robotic thyroidectomy and neck dissection *via* BABA are feasible in terms of surgical completeness, surgical safety, and oncological safety. The learning curve for working space making, robotic lobectomy, and total thyroidectomy are approximately 30, 20, and 20 cases, respectively. Our results provide a criterion for judging whether a surgeon has entered the stable stage of BABA RT in terms of the operative time. The reduction in complications does not need to reach the learning curve when performed by surgeons with prior endoscopic experience.

## Data availability statement

The original contributions presented in the study are included in the article/supplementary material. Further inquiries can be directed to the corresponding authors.

## Ethics statement

The studies involving human participants were reviewed and approved by Medical Ethics Committee of Central South University. The patients/participants provided their written informed consent to participate in this study. Written informed consent was obtained from the individual(s) for the publication of any potentially identifiable images or data included in this article.

## Author contributions

All authors made substantive intellectual contributions to this study to qualify as authors. FX conceived of the design of the study. XL modified the design of the study. HO, WX, RC, and ZZ performed the study, collected the data, and contributed to the design of the study. XL, BS, and HO analyzed the data. HO, WX drafted Result, Discussion, Conclusion sections. XL and FX drafted Methods sections. FX and XL edited the manuscript. All authors read and approved the final manuscript. All authors have agreed to be accountable for all aspects of the work in ensuring that questions related to the accuracy or integrity of any part of the work are appropriately investigated and resolved.

## Funding

This work was funded by the National Natural Science Foundation of China (82073262) and Natural Science Foundation of Hunan province (2019JJ4047).

## Conflict of interest

The authors declare that the research was conducted in the absence of any commercial or financial relationships that could be construed as a potential conflict of interest.

## Publisher’s note

All claims expressed in this article are solely those of the authors and do not necessarily represent those of their affiliated organizations, or those of the publisher, the editors and the reviewers. Any product that may be evaluated in this article, or claim that may be made by its manufacturer, is not guaranteed or endorsed by the publisher.

## Footnotes

1 The authors have completed the TREND reporting checklist.
